# Comparison of Experimental Middle East Respiratory Syndrome Coronavirus Infection Acquired by Three Individual Routes of Infection in the Common Marmoset

**DOI:** 10.1128/jvi.01739-21

**Published:** 2022-02-23

**Authors:** Michelle Nelson, Lyn M. O’Brien, Carwyn Davies, Emma Keyser, Wendy Butcher, Sophie J. Smither, Alejandro Nunez, F. Javier Salguero, M. Stephen Lever

**Affiliations:** a CBR Division, Defence Science and Technology Laboratory (Dstl), Porton Down, Salisbury, Wiltshire, United Kingdom; b Animal and Plant Health Agency, Weybridge, Addlestone, Surrey, United Kingdom; c Public Health England, Salisbury, United Kingdom; Loyola University Chicago

**Keywords:** animal model, Middle East respiratory syndrome, coronavirus, *Callithrix jacchus*, neutrophils, macrophages, aerosols, lung disease, interstitial pneumonia

## Abstract

Two strains of Middle East respiratory syndrome coronavirus (MERS-CoV), England 1 and Erasmus Medical Centre/2012 (EMC/2012), were used to challenge common marmosets (Callithrix jacchus) by three routes of infection: aerosol, oral, and intranasal. Animals challenged by the intranasal and aerosol routes presented with mild, transient disease, while those challenged by the oral route presented with a subclinical immunological response. Animals challenged with MERS-CoV strain EMC/2012 by the aerosol route responded with primary and/or secondary pyrexia. Marmosets had minimal to mild multifocal interstitial pneumonia, with the greatest relative severity being observed in animals challenged by the aerosol route. Viable virus was isolated from the host in throat swabs and lung tissue. The transient disease described is consistent with a successful host response and was characterized by the upregulation of macrophage and neutrophil function observed in all animals at the time of euthanasia.

**IMPORTANCE** Middle East respiratory syndrome is caused by a human coronavirus, MERS-CoV, similar to severe acute respiratory syndrome coronavirus 2 (SARS-CoV-2). Humans typically exhibit fever, cough, shortness of breath, gastrointestinal issues, and breathing difficulties, which can lead to pneumonia and/or renal complications. This emerging disease resulted in the first human lethal cases in 2012 and has a case fatality rate of approximately 36%. Consequently, there is a need for medical countermeasures and appropriate animal models for their assessment. This work has demonstrated the requirement for higher concentrations of virus to cause overt disease. Challenge by the aerosol, intranasal, and oral routes resulted in no or mild disease, but all animals had an immunological response. This shows that an appropriate early immunological response is able to control the disease.

## INTRODUCTION

Middle East respiratory syndrome is caused by a human coronavirus, MERS-CoV (Middle East respiratory syndrome coronavirus), which is a positive-sense, single-stranded RNA virus. Zoonotic human infection is associated with direct or indirect contact with dromedary camels, although human-to-human spread of the disease is common (1). The disease spreads via infected droplets or by touching infected surfaces (1). The first outbreak in humans occurred in 2012 in Saudi Arabia; however, this was subsequently traced back to an index case in Jordan (2). Humans typically present with fever, cough, shortness of breath, gastrointestinal issues, and breathing difficulties, which can lead to pneumonia and/or renal complications. By the end of June 2021, the World Health Organization (WHO) reported 2,574 human cases of Middle East respiratory syndrome, with a case fatality rate of approximately 34.4%. Unlike the closely related disease caused by severe acute respiratory syndrome coronavirus (SARS-CoV), MERS-CoV infection continues to persist in Middle Eastern countries and is a potential global threat. The WHO has identified it as a reportable infection. Consequently, there is a need for medical countermeasures to treat and prevent the disease. Hence, there is a requirement for suitable animal models to assess appropriate therapies and vaccines.

A number of animal species have been assessed to model human MERS-CoV infection, including rabbits, ferrets, Syrian hamsters, and mice (3–6), but found to be nonsusceptible to the virus. This was due to the function and distribution of the dipeptidyl peptidase 4 (DPP4) receptor, which is involved in attachment and cell entry (7). The structure of the receptor is diverse but is conserved in humans and nonhuman primates (NHPs), making nonhuman primates an ideal model of infection. The DPP4 receptor from a small nonhuman primate, the common marmoset (Callithrix jacchus), is predicted to have significant homology with the human receptor (8), unlike many of the animal models that have been used to assess the virulence of the virus. Susceptibility can be induced in mice, for example, by the transgenic expression of human DPP4 (9, 10). MERS-CoV has previously been studied in nonhuman primates, specifically rhesus macaques and marmosets (8, 11–15). Generally, marmosets appear to be more susceptible to the virus, with severe pneumonia being evident following challenge by multiple routes of infection simultaneously.

In this study, the virulence of two strains of MERS-CoV by three individual challenge routes, oral, intranasal, and aerosol, was determined. The two strains of MERS-CoV used in these studies were the Erasmus Medical Centre/2012 (EMC/2012) strain and the England 1 strain. EMC/2012 was isolated from the sputum of an infected individual early in the outbreak of MERS-CoV and was lethal (2). England 1 was isolated from a UK patient (16). This individual was seriously ill; however, this was not a fatal infection.

## RESULTS

### BHK cells transfected with either the human or marmoset DPP4 sequence facilitate the uptake of MERS-CoV into cells.

**FIG 1:**
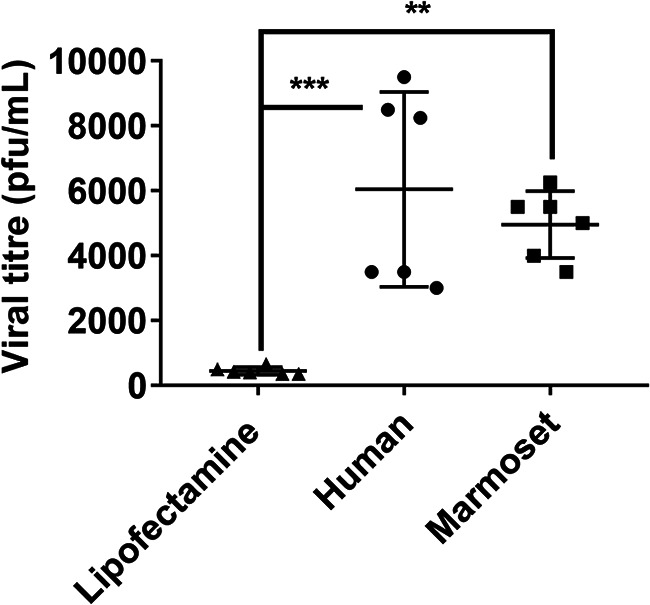
Viral replication of MERS-CoV in BHK cells following transfection with either human or marmoset DPP4 receptor RNA. Significant differences were determined by ANOVA, with differences shown between mock-transfected cells (Lipofectamine) and human and marmoset transfected cells (**, *P* < 0.01; ***, *P* < 0.001).

On two occasions, BHK cells were transfected with either the human or marmoset DPP4 sequence or mock transfected with Lipofectamine for 24 h. Mock-infected cells did not facilitate the replication of MERS-CoV strain EMC/2012. However, between 3 × 10^3^ and 9.5 × 10^3^ PFU/mL of virus were recovered from BHK cells transfected with human DPP4, whereas between 4 × 10^3^ and 6.3 × 10^3^ PFU/mL of virus were recovered from marmoset DPP4-transfected cells (Fig. 1). Both of these levels were significantly higher than those in mock-infected cells, suggesting that both marmoset and human cells allow the uptake of virus through the DPP4 receptor.

### Medium relative humidity with the virus collected into impinger fluid with 2% fetal calf serum was the optimal aerosolization condition.

**FIG 2:**
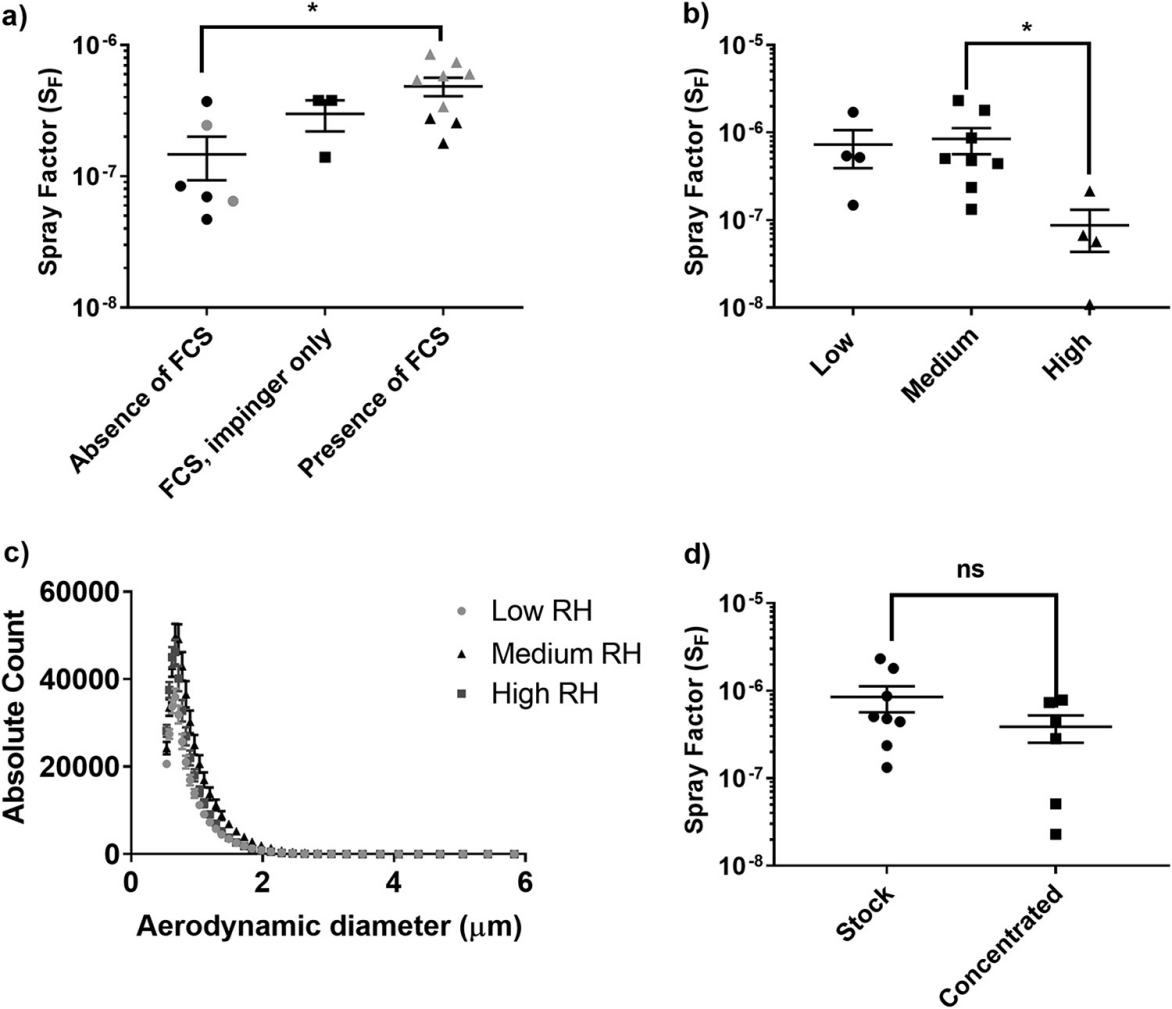
Optimization of the aerosolization of MERS-CoV. (a) Comparison of S_F_ values following the addition of 2% fetal calf serum (FCS) to the Collison and impinger fluids. (b) Comparison of the S_F_ values at different relative humidities, where low is 34.1% ± 2.2%, medium is 52.6% ± 3.6%, and high is 85.4% ± 6.0%. (c) Particle size distribution at different relative humidities. (d) Effect of ultracentrifugation and concentration of the viral stock on S_F_ values. Significant differences were determined by ANOVA (a and b) or a *t* test (d) with data transformed, *Y* = log(*Y*), to ensure normal data (ns, not significant [*P* > 0.05]; *, *P* < 0.05).

Prior to performing *in vivo* studies, it was necessary to determine the appropriate aerosolization conditions for the virus. Initially, three concentrations (approximately 1 × 10^5^, 1 × 10^6^, and 1 × 10^7^ PFU) of two strains of MERS-CoV, England 1 and EMC/2012, were aerosolized using a 3-jet Collison nebulizer, and the effect of the addition of 2% fetal calf serum (FCS) to the Collison nebulizer fluid and/or the impinger collection fluid was assessed. The spray factor (S_F_) was used to assess any difference between the conditions (Fig. 2a). A nonsignificant improvement in the efficiency of recovery and S_F_ was observed with the inclusion of 2% FCS in the impinger fluid only. The S_F_ increased from 1.47 × 10^−7^ ± 5.38 × 10^−8^ in the absence of 2% FCS to 2.99 × 10^−7^ ± 7.97 × 10^−8^ in the presence of 2% FCS in the impinger fluid. A further improvement was observed with the presence of 2% FCS in both the impinger and Collison fluids, with an S_F_ of 4.86 × 10^−7^ ± 7.80 × 10^−8^ (*P* = 0.0151 compared with no FCS). Due to potential concerns regarding *in vivo* complications associated with the delivery of FCS to animals, FCS was excluded from the Collison nebulizer fluid and used only in the impinger collection fluid.

The aerosolization of MERS-CoV strain EMC/2012 at a relative humidity (RH) of 85.4% ± 6.0% (“high”) had a detrimental effect on viral recovery and, therefore, the S_F_ (*P* = 0.0387) compared to a “medium” RH of 52.6% ± 3.6% (Fig. 2b). Additionally, the mass-median aerodynamic diameter (MMAD) of viral particles produced at high RH was reduced to 0.90 ± 0.24 μm, compared to 1.23 ± 0.03 μm and 1.19 ± 0.17 μm under medium- and low-RH (34.1% ± 2.2%) conditions, respectively (*P* = 0.002 and *P* < 0.0001 compared to low and medium RH, respectively) (Fig. 2c). Therefore, as there was no difference in the S_F_ or MMAD of particles generated under medium- and low-RH conditions, medium RH was selected for future studies, and this is easier to maintain in the laboratory.

A further assessment was performed to determine any effect of concentration of viral stocks on the S_F_. Concentration of viral stocks was required to increase the titer for *in vivo* studies and did not result in a significant change in the S_F_ (Fig. 2d).

### Pairs of marmosets were successfully challenged with two strains of MERS-CoV by individual routes of challenge.

Two studies were performed to assess the susceptibility of common marmosets to MERS-CoV by a single route of challenge. In study 1, pairs of marmosets were challenged by one of three challenge routes (aerosol, intranasal, or oral) with one of the two MERS-CoV strains (EMC/2012 or England 1). Animals received intranasal doses of 9.5 × 10^5^ PFU and 6.6 × 10^5^ PFU of MERS-CoV strains EMC/2012 and England 1, respectively (Table 1). Animals received oral doses of 9.5 × 10^6^ PFU and 6.6 × 10^6^ PFU of MERS-CoV strains EMC/2012 and England 1, respectively. The aerosol doses were calculated based on the volume of air that each animal received. Therefore, animals received a dose of either 3.2 × 10^3^ PFU or 2.4 × 10^3^ PFU of MERS-CoV strain EMC/2012 or 2.2 × 10^3^ PFU or 1.4 × 10^3^ PFU of MERS-CoV strain England 1.

**TABLE 1:** Details of marmosets used to assess the virulence of two strains of MERS-CoV by different routes of challenge^*a*^

Study and marmoset ID	Challenge strain	Challenge route	Challenge dose (PFU)	Time to fever (h)	Duration of fever (h)
Study 1					
91W	EMC/2012	Aerosol	3.20 × 10^3^	47.2	20.1
55W			2.40 × 10^3^	56.8	11.0
22X		Intranasal	9.50 × 10^5^	328.8	43.2
46X			9.50 × 10^5^	NA	NA
14X		Oral	9.50 × 10^6^	NA	NA
110W			9.50 × 10^6^	NA	NA
56W	England 1	Intranasal	6.60 × 10^5^	NA	NA
63W			6.60 × 10^5^	NA	NA
27X		Oral	6.60 × 10^6^	NA	NA
30X			6.60 × 10^6^	NA	NA
48X		Aerosol	2.20 × 10^3^	NA	NA
137X			1.40 × 10^3^	310	30

Study 2					
35X	EMC/2012	Aerosol	3.45 × 10^5^	26.8	41.4
29X			2.30 × 10^5^	28.0	40.7
130X			1.11 × 10^6^	21.6	25.1^*b*^
146X			4.42 × 10^5^	21.6	27.1^*b*^

a NA, not applicable.

b Euthanized during fever for a scheduled time point.

In study 2, two pairs of marmosets were challenged with a higher concentration of MERS-CoV strain EMC/2012 by the aerosol route only to determine whether disease severity increased with the challenge dose. Animals received between 2.3 × 10^5^ and 1.1 × 10^6^ PFU (mean dose of 5.3 × 10^5^ PFU) (Table 1).

### Higher doses of MERS-CoV strain EMC/2012 resulted in an earlier onset of fever.

**FIG 3:**
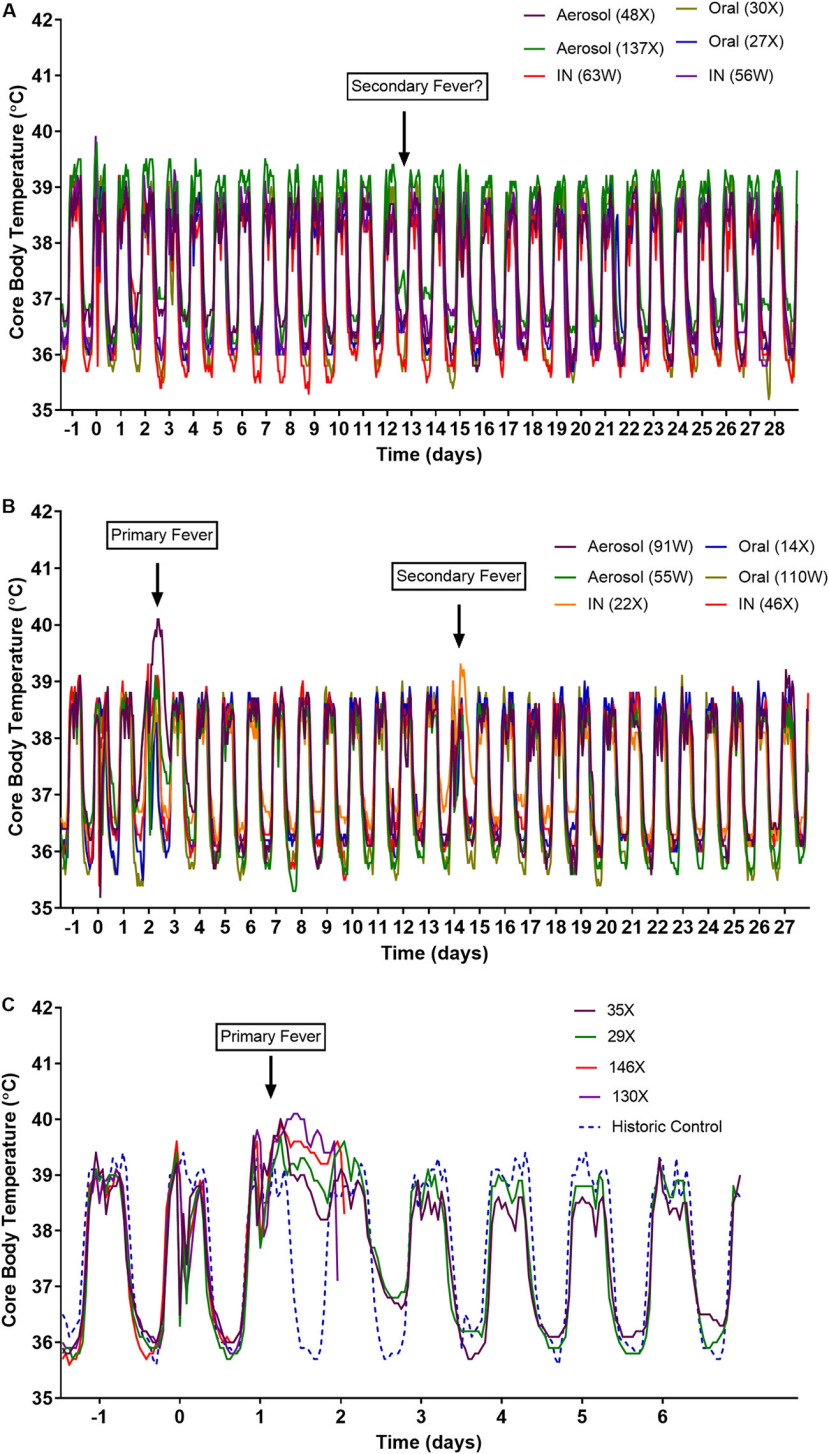
(A and B) Temperature profiles of marmosets following challenge with the original stocks of MERS-CoV strains England 1 (A) and EMC/2012 (B) by various routes of infection. (C) Temperature profile of marmosets following challenge by the aerosol route with the concentration stock of MERS-CoV strain EMC/2012.

Generally, all animals maintained their normal diurnal rhythm for the duration of the study (Fig. 3A and B). However, overnight on day 2 into day 3, the pair of animals challenged with the lower doses of EMC/2012 by the aerosol route defected from their normal diurnal rhythm (Fig. 3B). One of these animals (55W) returned to normal during the daytime phase. However, the other animal (91W) remained febrile until the morning of day 4. Higher challenge doses of MERS-CoV strain EMC/2012 by the aerosol route resulted in earlier, more pronounced fever in all animals by 28 h postchallenge (Fig. 3C). Animals 35X and 29X became febrile at 26.8 and 28 h postchallenge, respectively (Fig. 3C). They maintained their fever overnight, but by day 2, their daytime temperature had returned to normal. However, during the following nighttime, their temperature remained high, although the diurnal rhythm was observed. By day 3, their temperature profile had returned to normal, and the durations of the fever were 41.4 and 40.7 h for animals 35X and 29X, respectively. The other two animals, 130X and 146X, became febrile at 21.6 h postchallenge and remained febrile overnight. They were then euthanized, as scheduled, to assess pneumonia and viral dissemination at peak disease. By the time of euthanasia, they had fever for 25.1 h (animal 130X) and 27.1 h (animal 146X). No clinical signs were observed for any animal during the course of the study; however, there was a 5% decrease in the body weight of animals challenged with a high dose of virus by the aerosol route between days 2 and 4 postchallenge. A reduction in the levels of activity was observed on day 2 for the animals challenged with the lower dose of EMC/2012 by the aerosol route. No signs of respiratory distress were observed in any animal.

### Transient, delayed fever was apparent at approximately 13 days postchallenge.

An animal challenged with EMC/2012 by the intranasal route (animal 22X) had a transient fever starting overnight on days 13 to 14, reaching a temperature of at least 1°C above normal during day 14 (Fig. 3B). A further animal that had been challenged with England 1 by the aerosol route (animal 137X) had a potential secondary fever on two consecutive nighttime periods between day 12 and day 14, where the temperature was at least 1°C above normal (Fig. 3A). Pyrexia was not observed in any other animal.

### Viable virus was detected by a plaque assay in the lungs and throat swabs of some animals.

**FIG 4:**
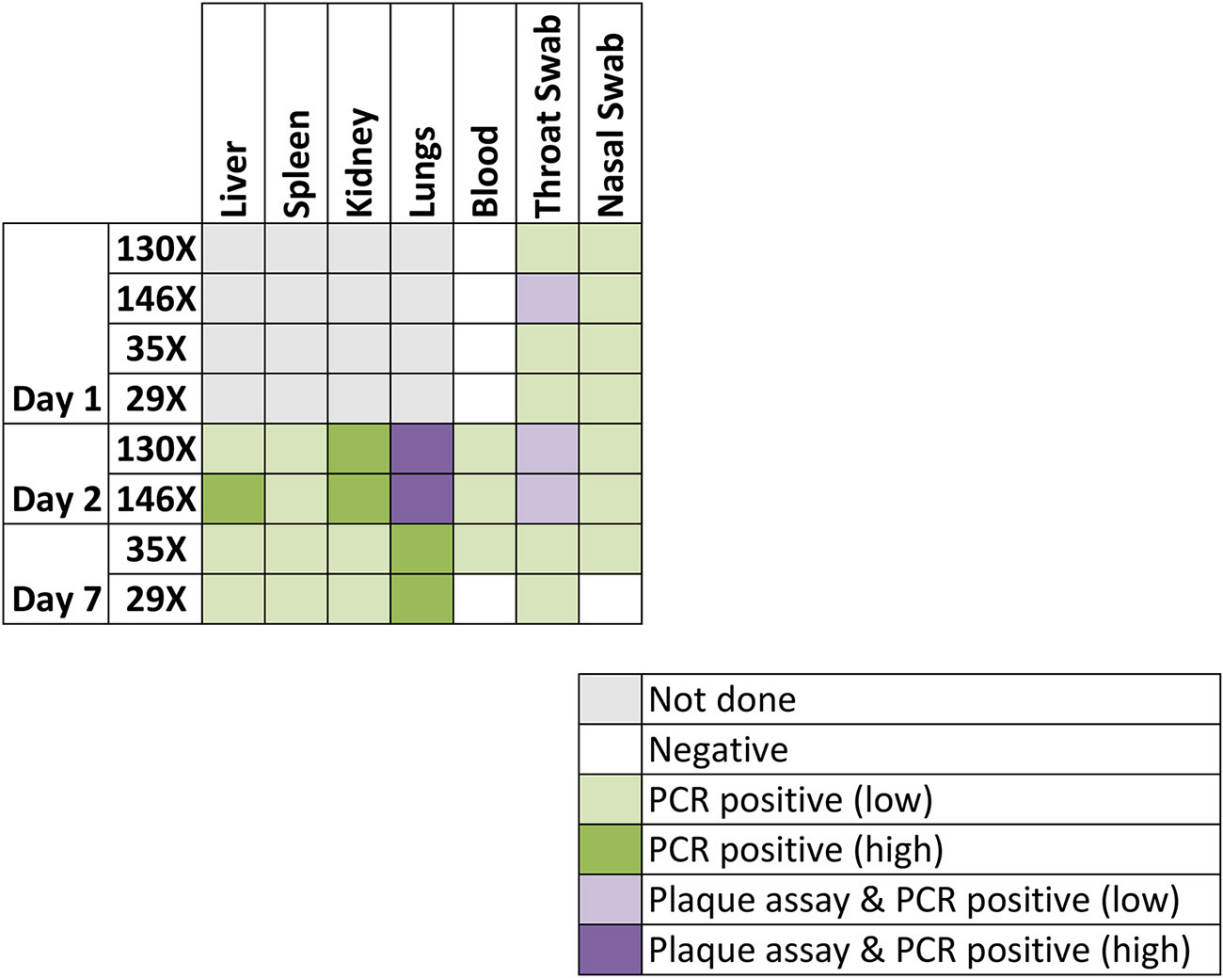
Heat map comparing the detection of virus by a plaque assay or PCR in tissues and swabs of marmosets challenged by the aerosol route with the concentration stock of MERS-CoV strain EMC/2012. Levels of viable virus are indicated in purple, with light purple indicating samples where <20 PFU of virus were identified and dark purple indicating samples where either 1.4 × 10^4^ or 7.7 × 10^4^ PFU were detected. Viral RNA was identified in all samples that were positive for viable virus, with between 1.1 × 10^2^ and 1.2 × 10^2^ PFU equivalents detected in light purple areas and between 4.1 × 10^6^ and 3.7 × 10^6^ PFU equivalents detected in dark purple areas. Viral RNA was also detected in other samples; light green indicates samples where <20 PFU equivalents were detected, and dark green indicates samples where between 1.1 × 10^3^ and 9.0 × 10^4^ PFU equivalents were detected.

Blood, tissue, and/or throat and nasal swabs were assessed for viable virus by a plaque assay and for nucleic acid by PCR. Both animals euthanized on day 2 (130X and 146X) had up to 7.67 × 10^4^ PFU of virus in their lungs (Fig. 4). Viable virus (<20 PFU) was also detected in the throat swabs of animal 146X, collected on day 1 and at the time of euthanasia (day 2). Viral nucleic acid was detected in these samples, and viral dissemination was evident, with viral nucleic acid being detected in the liver, spleen, kidneys, and day 1 blood samples. Additionally, viral nucleic acid was detected in throat and nasal swabs collected from both animals on day 1 and at the time of euthanasia. There was no evidence of viable virus or viral nucleic acid in the blood of any animal euthanized at day 7 or day 28. However, blood from one animal (55W) challenged by the aerosol route with strain EMC/2012 was positive for viral nucleic acid on day 14. Interestingly, low levels of viable virus (<20 PFU/g of tissue) were detected in the lungs of two animals (56W and 63W) challenged with the England 1 strain by the intranasal route and euthanized on day 28.

### Minimal histopathological changes were observed in the lungs of animals challenged with MERS-CoV by the intranasal and oral challenge routes, with occasional random foci of infection.

**FIG 5:**
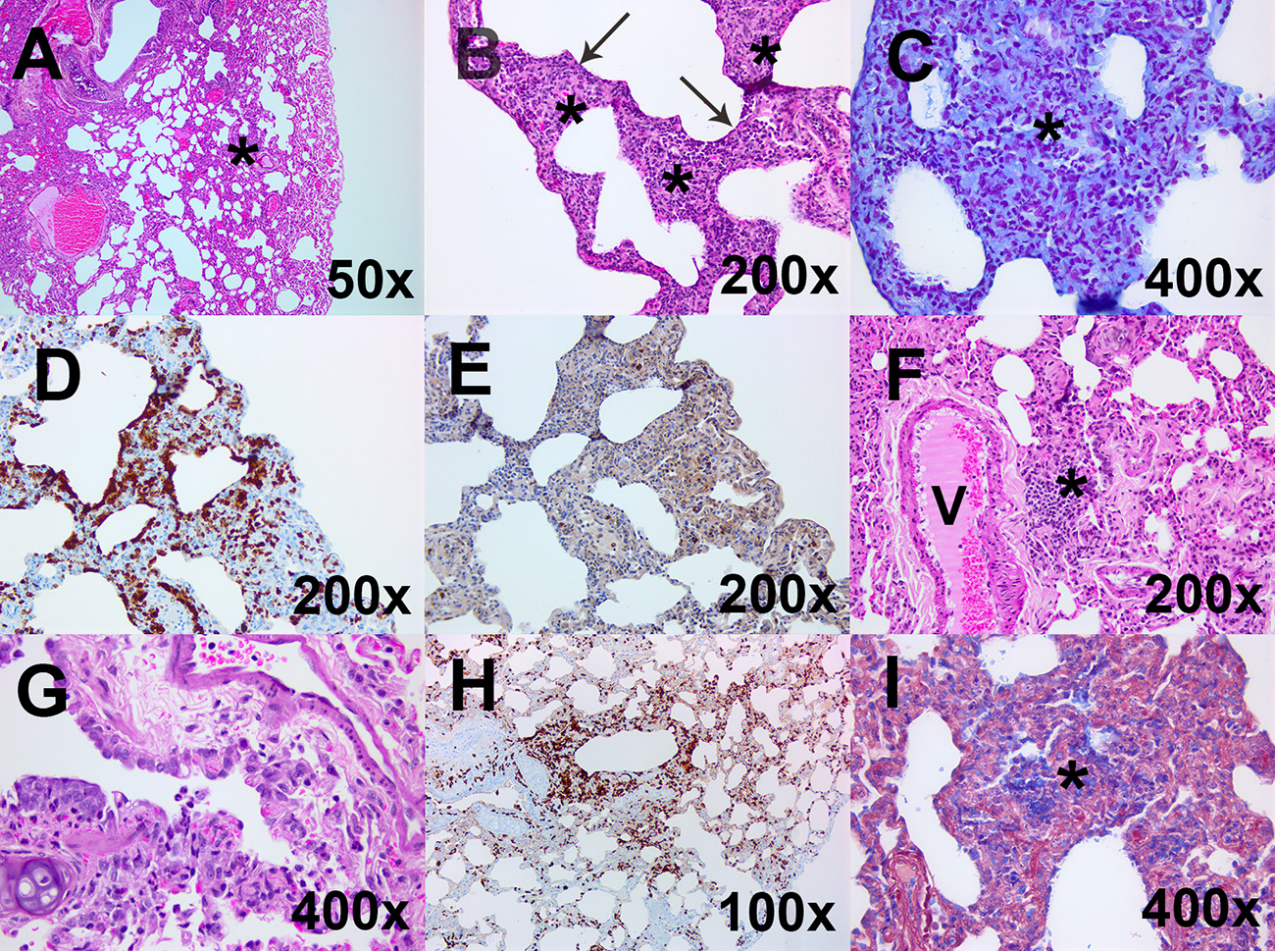
Representative histological images from the lungs of marmosets challenged by one of two strains of MERS-CoV by different routes of challenge. (A) A low-magnification hematoxylin and eosin (H&E)-stained image from animal 110W following challenge with strain EMC/2012 by the oral route shows mild thickening of alveolar septa and lymphocytic infiltration into the perivascular location (*) (above normal limits). (B) A high-magnification H&E-stained image from animal 56W following challenge with strain England 1 by the intranasal route shows expanded alveolar septa (*) infiltrated by neutrophils with mild exudation into the alveolar spaces (arrow). (C) This tissue was further characterized by Gram Twort staining and shows no evidence of bacterial colonies in areas of neutrophilic exudation. (D and E) Immunohistochemical staining with MAC387 shows an increase of cells in the alveoli (D), and immunohistochemical staining of CD3 shows scattered T lymphocytes in areas of alveolar thickening of septa (E). (F) A high-magnification H&E-stained image from animal 55W following challenge with strain EMC/2012 by the aerosol route shows lymphocytic infiltration into the perivascular location (*) (above normal limits). (G) H&E staining of the terminal bronchiole from animal 146X euthanized 2 days following challenge with the concentrated stock of MERS-CoV by the aerosol route shows exudation. (H) This sample was further characterized by immunohistochemical staining with MAC387 and shows an accumulation of cells in lesioned areas with exudation and septal infiltration. (I) Phosphotungstic acid hematoxylin (PTAH) staining was also performed on a lung section of the other animal euthanized 2 days following challenge with the concentrated stock of MERS-CoV (animal 130X) to show fibrin in the alveolar exudation.

Minimal pathological features were observed in animals challenged by the oral or intranasal route with either MERS-CoV strain EMC/2012 or England 1, following euthanasia on day 28 (Table 2). One animal (110W) challenged with MERS-CoV strain EMC/2012 by the oral route exhibited multifocal, mild thickening of the alveolar septa and lymphocytic infiltration in the perivascular location (Fig. 5A). A small focus of acute inflammation was observed in the lung of one animal challenged with MERS-CoV strain England 1 by the intranasal route (Fig. 5B). An area of expansion in the alveolar septa was observed, with infiltration of neutrophils and a mild exudation into the alveolar spaces. Interestingly, viable virus had been detected in a separate lung section from this animal, so the lesion was further characterized by immunohistochemistry (IHC). Gram Twort staining was performed to rule out the presence of a nonspecific bacterial response, although the area was associated with intense MAC387 staining and low levels of CD3 staining (Fig. 5C to E).

**TABLE 2:** Summary of the histological findings in the lungs of marmosets challenged with either of MERS-CoV strain England 1 or EMC/2012 by various routes^*a*^

Animal ID	Challenge route	Viral strain	Time of euthanasia postchallenge (days)	Severity of lung lesions
56W	Intranasal	England 1	28	Minimal to mild multifocal interstitial pneumonia
63W				Minimal to mild multifocal interstitial pneumonia
22X		EMC/2012	28	WNL
46X				Minimal multifocal interstitial pneumonia
27X	Oral	England 1	28	Minimal to mild multifocal interstitial pneumonia
30X				Minimal to mild multifocal interstitial pneumonia
14X		EMC/2012	28	WNL
110W				Mild multifocal interstitial pneumonia
48X	Aerosol	England 1	28	Mild multifocal interstitial pneumonia
137X				Mild multifocal interstitial pneumonia
91W		EMC/2012	28	Mild multifocal interstitial pneumonia
55W				Mild multifocal interstitial pneumonia
130X			2	Mild acute bronchointerstitial pneumonia
146X				Minimal acute bronchointerstitial pneumonia
35X			7	Mild subacute to chronic interstitial pneumonia
29X				Minimal subacute interstitial pneumonia

a WNL, within normal limits.

### The greatest pathological features were observed in animals challenged by the aerosol route with either MERS-CoV strain EMC/2012 or England 1.

In animals euthanized on day 28 postchallenge, the pathological features were mild and indicative of a chronic or resolving insult to the lungs. For all animals, thickening of the alveolar septa was observed, with lymphocytic infiltration into the septa and/or the perivascular location (Fig. 5F). Animals euthanized on day 2 postchallenge had more severe changes, exhibiting minimal to mild acute bronchointerstitial pneumonia. Multifocally, small groups of alveolar spaces appeared, occupied by a mixture of macrophages and neutrophils, small amounts of cell debris, and some fibrin (Fig. 5G). Degenerating cells are likely to be leukocytes and/or pneumocytes. Bronchi and bronchioles, especially terminal bronchioles, also displayed a mixed inflammatory exudation, and there were areas of epithelial attenuation, with bronchiolar cell degeneration and loss. Neighboring alveolar cells and those in perivascular locations appeared minimally to mildly expanded and infiltrated by histiocytic cells. In a small number of terminal bronchioles, there was mild histiocytic and neutrophilic exudation, with the presence of degenerating leukocytes, epithelial cells, and cell debris. This was indicative of cell death. Occasionally, in alveoli, the alveolar wall appeared indistinct with a loss of cellular definition of endothelial cells and pneumocytes, with the presence of cell debris and a few neutrophils in the air space, which may be pneumocyte necrosis. There was an increase in circulating neutrophils.

Animals euthanized on day 7 postchallenge had older, more chronic lesions suggestive of the reparation stage (data not shown). These animals had minimal to mild subacute to chronic interstitial pneumonia. Multifocally, the alveolar septa were expanded, thickened, and infiltrated by histiocytic cells and lymphocytes with a lymphohistiocytic exudation in the alveoli, with prominent alveolar macrophages with a large vacuolated cytoplasm. In these areas, type 2 pneumocytes were more numerous and prominent. Some perivascular and peribronchial spaces were also infiltrated by a similar cell population. Focally, in one animal, one of the thickened alveolar septa was expanded by connective tissue. Additionally, the mediastinal lymph node of this animal was active with increased cell trafficking in high endothelia and had expanded medullary sinuses with abundant lymph.

The liver, spleen, kidneys, lungs, various lymph nodes, and gastrointestinal tract were examined for morphological changes in animals euthanized on day 2 or 7 postchallenge. No disease-related pathological features were observed in any tissue other than the lung (data not shown).

### Acute, early lesions were associated with high levels of macrophages/neutrophils and moderate levels of T cells, both decreasing with time postchallenge.

The lesions in the lungs of animals euthanized on day 2 or 7 postchallenge were further characterized by immunohistochemistry. Moderate to diffuse levels of macrophages and neutrophils were identified by MAC387 staining in the lungs of the animals euthanized at day 2 (Fig. 5H). The cells were clustered in the bronchi, terminal bronchioles, and alveoli. There were also low to moderate levels of T cells, which were typically circulating in the areas around the lesions. B cells were rarely observed. Fibrin was evident in the alveolar and bronchial exudations of one animal, although none was detected in the lung section of the other animal (Fig. 5I). Less MAC387 staining was observed in the animals euthanized on day 7, with low to moderate levels being observed (data not shown). These were located in regions of thickened alveolar septa and peribronchial areas as well as in the exudation. Low to moderate levels of T cells were also associated with regions of thickened alveolar septa as well as an overall increase in connective tissue.

### MERS-CoV antigen was located in the same areas as the expression of the DPP4 receptor in the marmoset respiratory tract.

**FIG 6:**
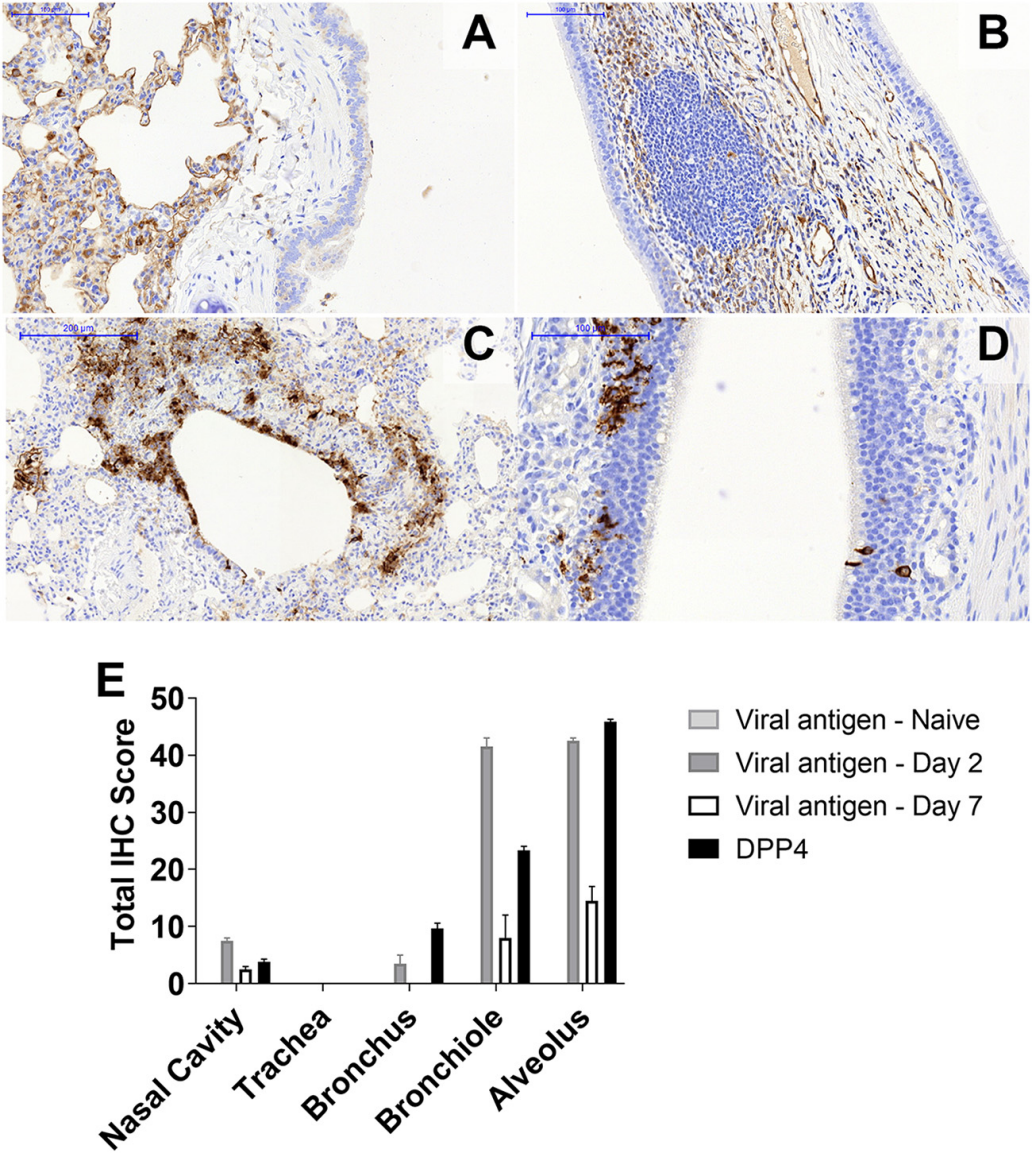
Location and activity of the DPP4 receptor in marmosets. (A and B) Immunohistochemical staining indicates a very strong presence of the DPP4 receptor within the alveolar spaces of marmosets (A) and no expression in the nasal cavity epithelium and moderate expression in the NALT and other submucosal structures (B). (C) The virus is observed in areas of high DPP4 receptor expression following aerosol challenge with MERS-CoV strain EMC/2012 within the terminal bronchioles and alveolar spaces. (D) An aggregate of MERS-CoV-positive lymphoid cells was observed in the nasal cavity. (E) Locations of MERS-CoV antigen and levels of expression of the DPP4 receptor in the respiratory tract and lungs of marmosets.

The respiratory tracts and the remaining lung sections from the animals euthanized on day 2 or 7 postchallenge were sectioned for further hematoxylin and eosin (H&E) staining and immunohistochemistry characterization. No significant histopathological changes were observed in the nasal cavity or other areas of the upper respiratory tract (including the larynx and trachea). The presence of activated NALT (nasal-associated lymphoid tissue) was detected with no biological significance. The presence of the DPP4 receptor in the tissues was very similar for all the animals. The expression level of DPP4 was very high in the alveolar spaces (Fig. 6A) and the terminal bronchioles, with little expression in the larger bronchi within the lung. The expression of DPP4 in the upper respiratory tract was very weak apart from some areas of the NALT and the submucosa (Fig. 6B).

The presence of MERS-CoV antigen was mainly observed in the alveoli and terminal bronchioles associated with areas of bronchointerstitial pneumonia (Fig. 6C). A low level of antigen was observed in the larger bronchi within the lung. The viral antigen was mainly associated with type 1 pneumocytes and, to a lesser extent, epithelial bronchiolar cells and macrophages. The presence of MERS-CoV antigen in the upper respiratory tract was limited to a few epithelial cells and sustentacular cells within the nasal epithelium (Fig. 6D). Low levels of antigen were also observed in some areas of the NALT, mostly within the caudal areas of the nasal cavity. No positive staining was observed in the sections of the larynx or the trachea from any animal.

The location of MERS-CoV antigen was greater in areas where there was a high expression level of the DPP4 receptor (Fig. 6E). The highest level of detection of viral antigen was observed in the alveoli and bronchioles in those animals euthanized at day 2 postchallenge. Although the absolute levels of the MERS-CoV antigen had decreased in those animals euthanized at day 7 postchallenge, there was relatively more viral antigen detected at that time in the alveoli and bronchioles.

### Evidence of liver dysfunction occurred early in the disease time course, with evidence of renal dysfunction later.

**FIG 7:**
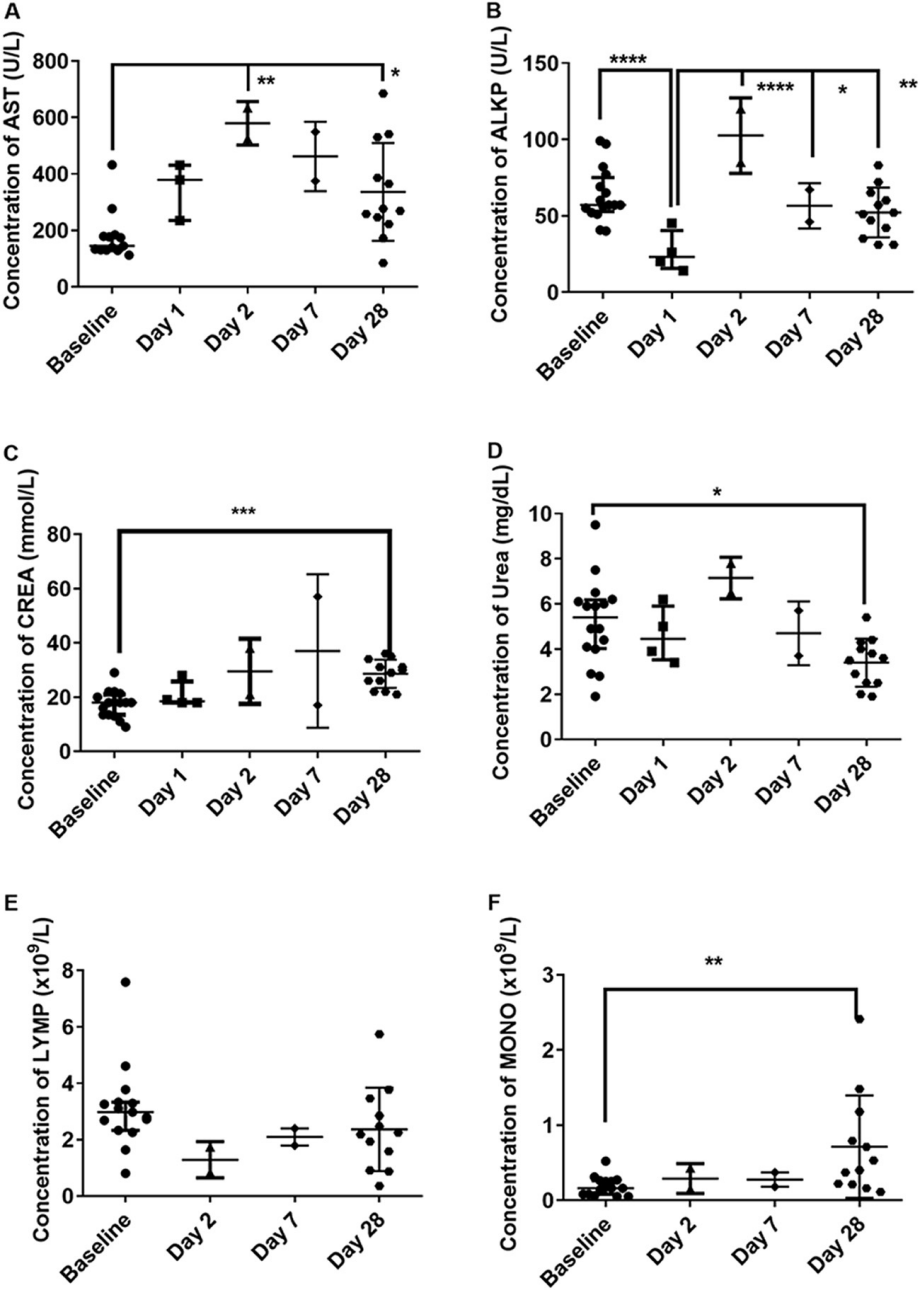
Clinical chemistry parameters observed in marmosets before and after challenge with two strains of MERS-CoV by various routes of challenge. (A) AST (aspartate aminotransferase); (B) ALKP (alkaline phosphatase); (C) CREA (creatinine); (D) BUN (blood urea nitrogen); (E) LYM (absolute lymphocyte count); (F) MONO (absolute monocyte count). For illustrative purposes, data from the two studies are combined on a single graph. Significant differences were determined by ANOVA with data transformed, *Y* = log(*Y*), to ensure normal data (*, *P* < 0.05; **, *P* < 0.01; ***, *P* < 0.001; ****, *P* < 0.0001).

Hematological and clinical chemistry parameters in the blood were compared prechallenge (baseline) and postmortem (Fig. 7). For illustrative purposes, the data from different experiments are depicted together on the graphs. Evidence of transient liver dysfunction was observed, with increasing levels of aspartate aminotransferase (AST) and alkaline phosphatase (ALKP) peaking in the animals euthanized at day 2 postchallenge (Fig. 7A and B). The levels of ALKP had returned to normal by 28 days postchallenge, although the levels of AST remained significantly high (*P* = 0.0312). Evidence of renal dysfunction was apparent with a general increase in the levels of creatinine (CREA), which was statistically significant at day 28 or 29 postchallenge (*P* = 0.0007) (Fig. 7C). This was associated with a statistically significant decrease in blood urea nitrogen (BUN) at day 28 or 29 postchallenge (*P* = 0.0432), with an initial increase being observed in animals euthanized at day 2 postchallenge (Fig. 7D). Consequently, there was a decrease in the BUN-to-CREA ratio. Further indicators of kidney dysfunction include a statistically significant increase in the uric acid (URIC) levels at day 28 or 29 postchallenge, a general increase in the levels of potassium (K) and magnesium (Mg), a decrease in the sodium-to-potassium (Na/K) ratio postchallenge, and a statistically significant increase in the osmolality calculation (Osm Calc) in animals euthanized at day 28 or 29 postchallenge (data not shown). The increased K, MG, and Osm Calc are also indicative of dehydration, although there was a reduction in water intake.

Overall trends showed a decrease in the red blood cell (RBC) count postchallenge, although this was not statistically significant (data not shown). However, there were some statistically significant changes in the RBC indices, particularly a decrease in the mean cell hemoglobin concentration (MCHC) and an increase in the mean corpuscular volume (MCV) (data not shown). This could be indicative of anemia, or the latter is also associated with liver dysfunction. A slight reduction in the number of lymphocytes was also observed, with an associated significant increase in the number of monocytes (*P* = 0.0057) (Fig. 7E and F).

### An early influx of CD54^+^ and CD80^+^ neutrophils occurred in the blood following challenge with MERS-CoV strain EMC/2012 by the aerosol route.

**FIG 8:**
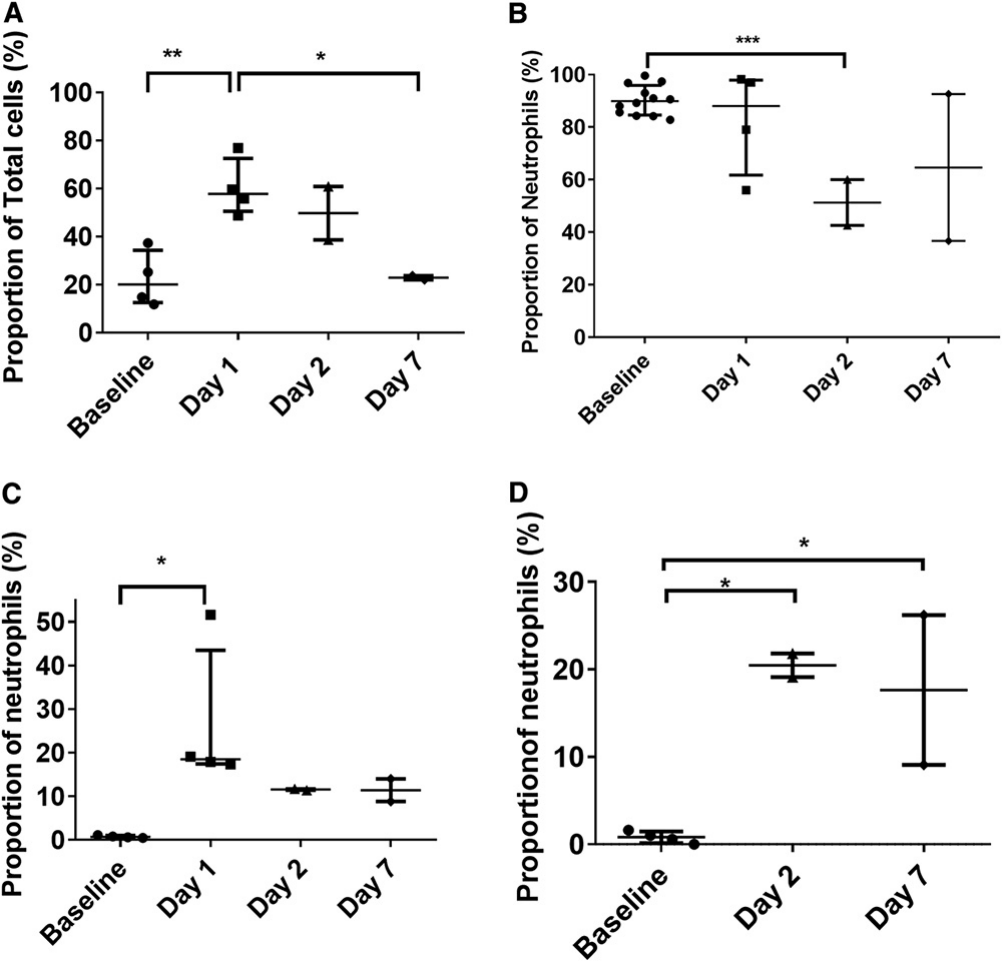
Proportions and activation status of different immune cells observed in marmoset blood following challenge with MERS-CoV strain EMC/2012 by the aerosol route. (A) Proportion of neutrophils; (B) HLA-DR expression of neutrophils; (C) CD54^+^ expression of neutrophils; (D) CD80^+^ expression of neutrophils. Significant differences were determined by ANOVA with data transformed, *Y* = log(*Y*), to ensure normal data (*, *P* < 0.05; **, *P* < 0.01; ***, *P* < 0.001).

The immunological response in the blood of animals challenged with MERS-CoV was assessed at various times postchallenge. The neutrophil proportion of white blood cells increased in animals challenged by the aerosol route from day 1 postchallenge before decreasing by day 7 (Fig. 8A). In conjunction with the increase in the neutrophil proportions, there was also a change in the activation status of various key markers: HLA-DR^+^, CD54^+^, CD80^+^, and CD16^+^ (Fig. 8B to D). HLA-DR^+^ expression decreases in animals at acute stages of disease and is a general health indicator for marmosets. The levels of HLA-DR^+^ expression had decreased in two out of the four animals that had blood collected on day 1 postchallenge and was significantly decreased in the animals euthanized on day 2 (*P* = 0.00060) (Fig. 8B). One of the animals euthanized on day 7 postchallenge also had decreased expression of HLA-DR^+^. CD54^+^ expression was increased in all animals between day 1 and day 7 postchallenge. The highest levels were observed at day 1 postchallenge (*P* = 0.0323). This is indicative of neutrophil migration to the tissues, and an increase in the proportion of neutrophils in the lungs was observed in one animal on day 2 (Fig. 8A). Similarly, CD80^+^ expression was increased in the animals euthanized on day 2 and day 7 postchallenge (*P* = 0.0207 and *P* = 0.0371, respectively). This may be indicative of the neutrophils interacting with T cells. The levels of CD16^+^ expression remained unchanged (data not shown). There was no change in the expression of CD54^+^ or CD80^+^ on the neutrophils of the animals that were challenged with either strain of MERS-CoV by various routes and euthanized on day 28 postchallenge.

There were minimal changes in the number of macrophages (data not shown); however, there was an increase in their activation status in these animals following challenge with MERS-CoV. This included a significantly high level of CD16^+^ expression on day 2/3 postchallenge compared to the baseline (*P* = 0.0055). CD40^+^ levels increased significantly at day 2 (*P* = 0.0382), while CD80^+^ expression increased significantly at day 7 (*P* = 0.0074). The activation status of T cells increased postchallenge (data not shown). There was increased expression of the early activation marker CD69^+^ at day 2 in one animal only, while elevation was observed in both animals euthanized on day 7. The expression of the γδ T-cell marker was also increased in these animals.

### Increased activation of T cells, neutrophils, and macrophages occurred in the lungs of marmosets following aerosol challenge.

**FIG 9:**
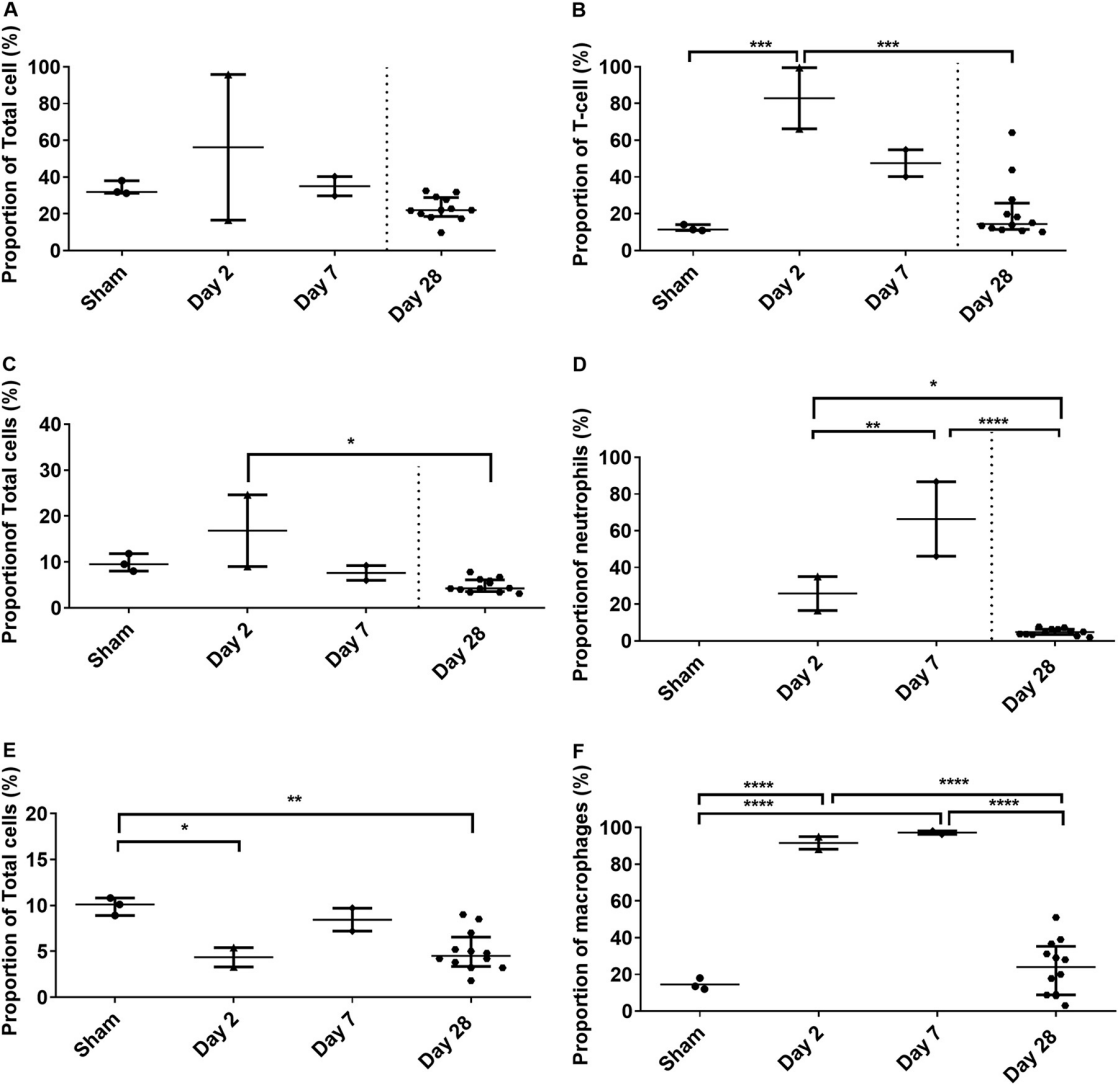
Proportions and activation status of different immune cells observed in marmoset lungs following challenge with MERS-CoV. (A) Number of T cells; (B) CD16^+^ expression of T cells; (C) proportion of neutrophils; (D) CD80^+^ expression of neutrophils; (E) proportion of macrophages; (F) CD40^+^ expression of macrophages. For illustrative purposes, levels are shown for animals challenged with MERS-CoV strain EMC/2012 by the aerosol route and euthanized on days 2 and 7. Significant differences were determined by ANOVA with data transformed, *Y* = log(*Y*), to ensure normal data (*, *P* < 0.05; **, *P* < 0.01; ***, *P* < 0.001; ****, *P* < 0.0001).

The immunological response was also assessed in the lungs of all animals at the time of euthanasia. The proportion of CD8^+^ T cells was increased in one animal euthanized at day 2 postchallenge (Fig. 9A). This was associated with a substantial increase in the expression of CD56^+^ (natural killer [NK] T cells) in that animal (data not shown). The expression of CD56^+^ was also increased in the other animal euthanized on day 2 as well as both animals euthanized on day 7. The expression of CD69^+^ and CD16^+^ was significantly increased in both animals euthanized on day 2 (*P* = 0.0419 and *P* = 0.0009, respectively), and CD16^+^ expression was also increased in both animals euthanized on day 7 (Fig. 9B). The proportions of neutrophils in the lungs remained similar in all animals except one animal euthanized at day 2 postchallenge (Fig. 9C). However, significantly increased expression of CD80^+^ (marker of communication with T cells) was observed in both animals euthanized on days 2 and 7 (*P* = 0.0222 and *P* < 0.0001, respectively). Similarly, the proportions of macrophages in the lungs remained similar in all animals, but high activation was observed in animals euthanized on days 2 and 7 (Fig. 9E and F). Increased expression was observed in both animals euthanized on days 2 and 7 for CD40^+^ (classical macrophages) (*P* < 0.0001 for both), CD16^+^ (unbiased activation marker) (*P* = 0.0007 and *P* < 0.0001, respectively), and CD54^+^ (migratory marker) (*P* = 0.0271 and *P* = 0.0119, respectively).

### Cytotoxic CD8^+^ T cells and natural killer cells are activated 4 weeks after subacute to mild infection with MERS-CoV.

**FIG 10:**
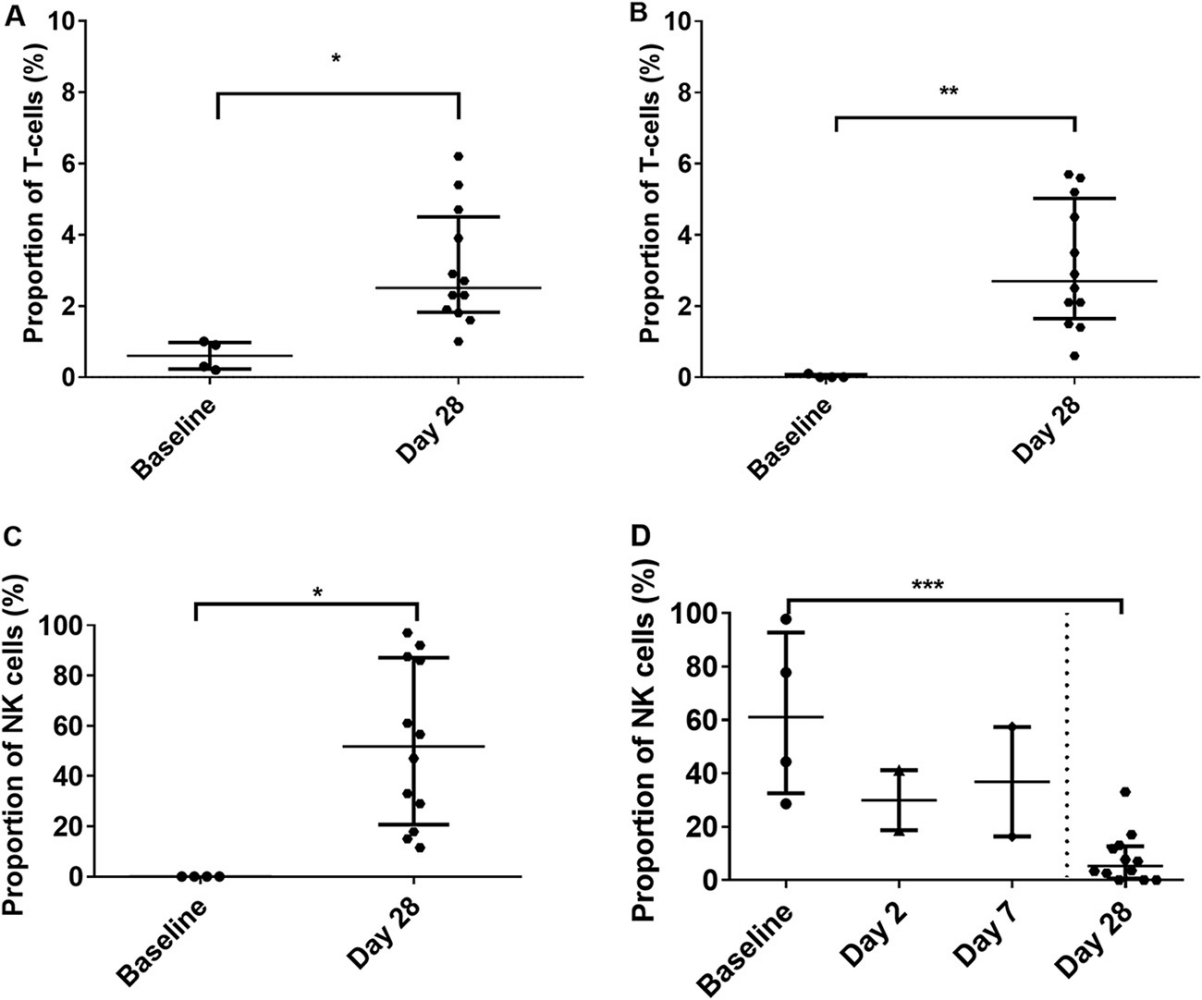
Activation status of different immune cells observed in marmoset blood following challenge with either MERS-CoV strain EMC/2012 or England 1 by different routes of challenge. (A) Cytotoxic CD8^+^ cells; (B) NK^+^ T cells; (C) CD69^+^ expression of NK cells; (D) CD16^+^ expression of NK cells. For illustrative purposes, expression levels of CD16^+^ on NK cells are shown for animals challenged with MERS-CoV strain EMC/2012 by the aerosol route and euthanized on days 2 and 7. Significant differences were determined by ANOVA with data transformed, *Y* = log(*Y*), to ensure normal data (*, *P* < 0.05; **, *P* < 0.01; ***, *P* < 0.001).

The proportions of upregulated cytotoxic T cells and NK^+^ T cells were significantly increased on day 28 regardless of whether overt disease was observed in the animals (*P* = 0.0113 and *P* = 0.0037, respectively) (Fig. 10A and B). The levels of CD69^+^ expression on natural killer cells varied between 11 and 97% expression but were significantly higher than the baseline levels (*P* = 0.0061) (Fig. 10C). This indicates a role in the proliferation of the cells as well as the stimulation of antibody-dependent cellular cytotoxicity. CD16^+^ expression on NK cells was increased in animals that were challenged by the aerosol route with a high dose of MERS-CoV strain EMC/2012 on day 2 and day 7 postchallenge (Fig. 10D). However, the levels had returned to baseline levels in animals that were euthanized on day 28. CD16^+^ and CD54^+^ expression was also observed on neutrophils in animals euthanized on day 28 (Fig. 10C and D). Lower levels of expression of all three markers were also observed on macrophages in the lungs of animals euthanized on day 28.

Plasma collected from animals euthanized on day 28 was used to measure the IgG antibody response to whole, live strain-specific coated plates. There was no antibody response observed in animals at day 14 or 15 postchallenge. However, 4 animals had low to moderate antibody titers (1:50 to 1:100) at day 28: 2 animals challenged with EMC/2012 and 2 animals challenged with England 1. The antibody-positive sera were then assessed for their levels of neutralizing antibodies, and 3 of the 4 achieved a ≥90% reduction (90% plaque reduction/neutralization titer [PRNT_90_]). The remaining animal (EMC/2012-challenged animal) achieved a >70% reduction (PRNT_50_) in the number of virus plaques at a 1:50 dilution.

## DISCUSSION

The aim of this work was to assess the virulence of two strains of MERS-CoV in the marmoset by three separate challenge routes to develop a nonhuman primate (NHP) model of infection for the assessment of medical countermeasures. The common marmoset (Callithrix jacchus) is a New World monkey (NWM) species that has been used as an alternative NHP model to complement the more traditionally used Old World monkeys (OWMs) (e.g., rhesus and cynomolgus macaques). In general, marmosets are reported to have greater susceptibility to MERS-CoV than rhesus macaques; typically, the disease ranges from moderate to severe in marmosets, while the disease in rhesus macaques is mild to moderate (11). Previous reports of experimental marmoset infection have indicated great variation in the disease presentations and outcomes. For example, severe disease with 22% lethality occurred following challenge with 5.2 × 10^6^ 50% tissue culture infective doses (TCID_50_) of the EMC/2012 strain by multiple routes of administration simultaneously (8). Alternatively, nonlethal disease with mild interstitial pneumonia occurred following challenge with 5 × 10^7^ PFU of either the Jordan-n3/2012 or EMC/2012 strain intratracheally (i.t.) (12).

Three routes of infection were used in this study. Two routes of infection, the intranasal and oral routes, had previously been investigated in combination with other routes (8). However, MERS-CoV delivered by the aerosol route had not previously been investigated in marmosets. The aerosol route has been suggested as a possible mechanism of disease spread with viral RNA isolated from air collected in hospitals and in barns containing camels (17, 18). Aerosolization conditions were optimized in this study such that medium relative humidity (50 to 55%) facilitated the delivery of small particles and had viral recovery similar to that with low relative humidity (30 to 35%). Previous work suggested that MERS-CoV was more stable at low temperatures and low relative humidity (20°C and 40%) and that high relative humidity (70%) had a detrimental effect on viral recovery (19). The work reported in this study was performed at 23°C. However, a similar trend was observed with both groups determining that high relative humidity was detrimental to viral recovery. During the optimization studies, a reduction in viral recovery was observed at medium relative humidity when the temperature was increased to 25°C (data not shown).

The small-particle aerosols generated ranged from 1 to 3 μm and were deposited in the lower alveoli. Such deposition of aerosolized particles is optimum for MERS-CoV infection as it correlates with the reported location of the dipeptidyl peptidase 4 (DPP4) receptor in marmosets. DPP4 is the host receptor for the attachment of MERS-CoV, thus facilitating entry into the cell (20). The expression of the receptor in the lower respiratory tract is associated with more severe disease in humans and nonhuman primates. Expression in the upper respiratory tract of dromedary camels results in mild disease and these animals shedding the virus, leading to human infection. Immunohistochemistry identified the expression of the DPP4 receptor in marmosets to be predominately in the lower respiratory tract, with very high levels in the alveolar spaces and the terminal bronchioles. Limited expression was observed in the larger bronchi within the lung. In the upper respiratory tract, the expression level of the DPP4 receptor was low, with the exception of some areas of the NALT and the submucosa. A similar pattern of DPP4 expression was observed in marmosets challenged by the intratracheal route (21). These animals were euthanized on day 3 postchallenge during acute disease, and only acute lesions were observed. Edema was also observed in those animals, which was not evident in the animals challenged by the aerosol route.

Following aerosol challenge, MERS-CoV antigen was proportionally colocated with the expression of the DPP4 receptor. The viral antigen was mainly observed in the alveoli and terminal bronchioles associated with areas of bronchointerstitial pneumonia. Low levels of antigen were also observed in some areas of the NALT, mostly within the caudal areas of the nasal cavity. MERS-CoV appeared, therefore, to have been successfully taken up by the marmoset lung cells following aerosol challenge, but there was limited, if any, evidence of viral replication. This ability for the marmoset DPP4 receptor to act as a conduit to facilitate MERS-CoV uptake into cells was further demonstrated in the transfection assay. The similarity in the uptake of marmoset and human transfected cells in this assay is unsurprising as the sequence of marmoset DPP4 is 96.4% identical to that of the human receptor (8). Additional studies also confirmed that there were no sequence differences observed in the receptor binding domain (22).

Viable virus was isolated from host tissue as well as throat swabs of one animal challenged by the aerosol route with MERS-CoV strain EMC/2012 on days 1 and 2 postchallenge. Additionally, both animals euthanized on day 2 postchallenge had low levels of viable virus in their lungs. Previously, viable virus had not been isolated from nonhuman primates or humans. Viable virus had been obtained from nasal swabs of camelids (23), pigs (24), and rabbits (3), although no infectious virus was observed in any tissue. Of the two reported human autopsies, viral antigen was detected only in the lungs (25) or the lungs, renal tissue, and skeletal muscle (26) by immunohistochemistry.

The detection of viral nucleic acid was much more prevalent in marmosets challenged by the aerosol route: in the throat swabs from four animals on day 1 postchallenge and in the lungs of both animals on day 2 postchallenge. Higher PFU-equivalent values were obtained for the samples that were positive by the plaque assay. This may be due to the detection limit of the plaque assay, or it could be because PCR amplified nonintact and nonviable virus. This is notable in all other animal models as well as the few clinical cases where detection has been reported. Dissemination of virus was observed, with detection of RNA in the liver, spleen, and kidneys of all animals, with the highest numbers in the kidneys of animals euthanized on day 2 postchallenge, providing evidence of widespread dissemination. In addition, viable virus was isolated from the lungs of a further two animals that had been challenged by the intranasal route with MERS-CoV England 1 and euthanized on day 28 postchallenge.

The disease in marmosets shares typical features of the human disease, including renal and liver dysfunction (27–29). However, the disease presentation in the current study was less severe than that reported previously in marmosets when MERS-CoV was delivered via multiple challenge routes (8) and more comparable with the disease outcome when virus was delivered via the intratracheal route only (12). In all cases, varying severity of multifocal bronchointerstitial pneumonia was observed, typically associated with the bronchi, terminal bronchi, and alveoli. However, in this study, there was no evidence of edema that had been reported in marmosets. Only those animals challenged with MERS-CoV strain EMC/2012 by the aerosol route developed pyrexia, with an early febrile response associated with a higher challenge dose.

Intriguingly, there appears to be a potential second phase of disease, as two animals challenged in the original study had transient fever at around day 13 postchallenge. Blood was collected from the animals on day 14 or 15, and four animals had decreased neutrophil HLA-DR expression, including one of the animals with secondary transient fever. This marker has previously been demonstrated to be a sensitive marker of well-being in the marmoset, decreasing prior to the onset of clinical signs, including fever. This study is the first demonstration that the expression of HLA-DR was reduced in some animals that did not subsequently develop fever. HLA-DR expression has been shown to increase when the animal’s condition improves, e.g., upon treatment with antibiotics (M. Nelson, personal communication). One of the animals that had primary fever was also positive for RNA in the blood at this time. This animal was the only animal that was PCR positive for viral RNA in the lungs at the end of the study on day 28 postchallenge. At the end of the study, on day 28 postchallenge, five animals had decreased HLA-DR expression, including two animals that had not previously shown signs of illness. Therefore, MERS-CoV, for the routes and doses administered, caused mild, potentially undulating disease over a 28-day period. This may be due to a resurgence of MERS-CoV from small foci of viable virus that had been observed in the lungs of one animal.

Despite the lack of overt signs, there was further immunological evidence of disease. A transient decrease in the expression of HLA-DR on neutrophils was observed in nine animals that had been challenged by either the aerosol or intranasal route of exposure. It is likely that these animals had developed a subclinical response to the disease, which was controlled quickly by the host response. Indeed, there was an early innate response from day 2 postchallenge with increases in the proportions and activation status of neutrophils and macrophages. Levels of natural killer (NK) cells also increased on day 2, with this cell type being essential for the early immune response to viruses. Indeed, NK cells are the target cells for a range of virus infections (30) and are essential for viral clearance. The level of CD16^+^ expression on NK cells during the first 7 days of infection was reduced and remained low, which suggested increased NK function, as shedding of CD16^+^ is known to occur after activation (31). By day 28, the NK cells had high expression of CD69^+^, suggesting continued activation.

The disease following oral exposure was the most subclinical, with no clear readouts despite a robust immunological response on day 28 postchallenge, indicating that there was a good host response that kept the disease under control. Disease readouts were apparent for both the aerosol and intranasal routes of exposure, with more severe interstitial pneumonia, more consistent renal dysfunction, and pyrexia. Animals challenged with MERS-CoV strain EMC/2012 by the aerosol route had greater early innate responses. There was a more pronounced systemic increase in the numbers of neutrophils and macrophages. The macrophages were highly activated to deal with the insult, whereas the neutrophils were primed to migrate to the tissue and communicate with T cells.

In conclusion, despite the lack of clear overt disease, pyrexia was observed in animals challenged by the aerosol route with MERS-CoV strain EMC/2012 with evidence of a dose response. Viable virus was isolated from these animals, and there was evidence of viral dissemination. Additionally, the early innate response was sufficient to reduce the severity of the disease, and a primed immune response was still observed at 28 days postchallenge.

## MATERIALS AND METHODS

### Viral strains.

The MERS-CoV England 1 strain and EMC/2012 strain were supplied by the NCPV (catalog number 1409231v, lot number 1390) and BEI (catalog number NR-44260, lot lumber 62043787), respectively, and working stocks were prepared.

For the enumeration of virus, viable counts were performed by a plaque assay. Vero cells were adapted and maintained in serum-free medium and seeded onto 24-well plates at ≥1 × 10^5^ cells/mL. Tenfold serial dilutions of the virus were performed in antibiotic-supplemented serum-free medium, and 200 μL of each dilution was added to cells. Each sample was assayed in triplicate. The virus was left to absorb onto the cells for 1 h at 37°C. Carboxymethyl cellulose (CMC) was overlaid onto all wells, and the plates were incubated at 37°C for 4 days. The plates were then fixed with 20% formalin at room temperature (RT) overnight and stained with a 0.2% crystal violet solution to enable the counting of plaques.

### Virus concentration.

On six occasions, 10 flasks containing Vero cells supplemented with 4% FCS were infected with MERS-CoV strain EMC/2012 Working Cell Bank (W193) at a multiplicity of infection (MOI) of 0.0001. Flasks were incubated at 37°C for 3 days, and the supernatants were harvested when the observed cytopathic effect reached a minimum of 70% (approximately 72 h). Viral supernatants were clarified by centrifugation in a Beckman Coulter SW28 rotor at 3,000 rpm for 10 min. Virus was then purified by centrifugation through a 30% (wt/vol) sucrose cushion using the SW28 rotor at 25,000 rpm for 2.5 h at 4°C. Viral pellets were resuspended in a total of 2.4 mL Tris buffer (pH 7.2) and sonicated for 3 min.

### Transfection assay.

Transfection experiments with DPP4 were performed as previously described (32). In brief, the coding sequences of human and marmoset DPP4 (GenBank accession numbers NM_001935.3 and XM_002749392, respectively) were synthesized and inserted into pcDNA3.1 (Thermo Fisher Scientific). Baby hamster kidney cells (BHK-21), in a 24-well plate, were transfected with 0.5 mg of DNA using the Lipofectamine 2000 reagent (Life Technologies), according to the manufacturer’s instructions. The uniformity of expression of the receptor was assumed to be ubiquitous in these studies. After 24 h, transfected and mock-transfected cells were infected with MERS-CoV (EMC/2012) at an MOI of 1. The titer of the virus in the supernatants was determined after an incubation period of 48 h.

### Animals.

Healthy, sexually mature common marmosets (C. jacchus) were obtained from the Defence Science and Technology Laboratory, Porton Down (Dstl Porton Down), breeding colony and housed in vasectomized male and female pairs. The mean age and weight of the animals used were 2.7 ± 0.1 years and 443 ± 15 g at the time of challenge. All animals were allowed free access to food and water as well as environmental enrichment. All animals were surgically implanted intraperitoneally with a Remo 201 device (EMMS, Bordon, Hampshire, UK) under general anesthesia (ketamine/medetomidine [Domitor]/isoflurane) to record the core body temperature (*T_c_*). Prophylactic pain relief of meloxicam (0.1 mL/kg of body weight) and buprenorphine (0.005 mg/kg) was administered subcutaneously. After surgery 0.2 mg/kg of meloxicam was administered orally for 5 days. Temperature data were transmitted from the devices at 30-s intervals to locally placed antennas and relayed to receivers. Data were analyzed using eDacq software to provide real-time and recordable *T_c_* values (EMMS, Bordon, Hampshire, UK). The animal studies were carried out in accordance with the UK Animals (Scientific Procedures) Act of 1986 (35). Animals were allowed to recover from the surgical implantation for a minimum of 4 weeks prior to challenge. Following challenge, animals were handled under animal containment level 3 (CL3) conditions, within a half-suit isolator compliant with British Standard BS5726. Blood was collected from animals on day 1 (study 2) or day 2 or 3 (study 1) postchallenge and assessed for viral load and immunological parameters. Concurrently, nasal and throat swabs were obtained from the animals. The swab was cut, and the head of the swab was placed into 2 mL of virus dilution medium in a sealed bijoux.

### Intranasal challenge.

One hundred microliters of the neat suspension of the appropriate virus was instilled in a dropwise fashion into alternate nares of the animal sedated with 10 mg of ketamine hydrochloride by the intramuscular route.

### Oral challenge.

For oral administration, 1 mL of the appropriate neat suspension of the virus was added to 1 mL of banana-flavored Nesquik powder dissolved in water. The liquid was presented in a syringe to preconditioned animals to accept.

### Aerosol challenge.

Prior to challenge, animals were anesthetized with 10 mg/kg of ketamine hydrochloride via the intramuscular route. Briefly, an aerosol was generated using a 6-jet Collison nebulizer containing a 10-mL suspension of the appropriate concentration of the appropriate MERS-CoV strain using a contained Henderson apparatus controlled by the AeroMP (Aerosol Management Platform) system (Biaera Technologies LLC). Animals were placed within a plethysmography tube and attached to the exposure unit as previously described (33). Pairs of animals were exposed to the aerosol for 10 min via a head-only exposure chamber, with samples impinged from the chamber using an AGI-30 impinger (Ace Glass Inc., USA) containing phosphate-buffered saline (PBS) at 12 L/min. The accumulated volume of air breathed by each animal was determined in real time using eDacq software (version 1.8.4b). The dose that each animal received was calculated as follows: aerosol concentration (PFU/L of air) = [impinger count (PFU/mL) × impinger volume (mL)]/[impinger flow rate (L/min) × impinger time (min)], and dose received (PFU) = aerosol concentration (PFU/L of air) × total accumulated volume (L).

### Postmortem analysis.

Animals were euthanized at either 28 days postchallenge (study 1) or 2 or 7 days postchallenge (study 2), and organs were aseptically removed, weighed, and examined for gross pathological changes. For virology, weighed sections were homogenized into 1 mL of virus dilution medium, and enumeration of the viral load was performed as described above. In addition, the liver, spleen, kidneys, lungs, gastrointestinal tract, respiratory tract, and lymph nodes were placed into 10% neutral buffered formalin for histological analysis. In addition, blood was collected via postmortem cardiac puncture into sodium citrate (bacteriological and immunological analyses), lithium heparin (clinical chemistry analysis), and EDTA tubes (hematology analysis). Albumin, alanine aminotransferase (ALT), aspartate aminotransferase (AST), alkaline phosphatase (ALKP), amylase, total bilirubin, blood urea nitrogen, calcium, phosphate, total protein, creatine, and glucose levels were measured using a Catalyst Dx system (Idexx Laboratories). Hematology levels (red blood cells, white blood cells, hematocrit, platelets, neutrophils, monocytes, eosinophils, and basophils) were measured using a Procyte Dx system (Idexx Laboratories).

### PCR.

The QIAamp viral RNA minikit (Qiagen) was employed to extract RNA from 140 μL of homogenized organs or blood harvested from marmosets postchallenge, according to the manufacturer’s instructions. In order to generate standard curves for use in reverse transcription-PCR (RT-PCR), various amounts of MERS-CoV (EMC/2012) were added to homogenates of organs harvested from naive marmosets. A total of 140 μL of each suspension was extracted using the QIAamp viral RNA minikit to produce a standard curve ranging from 1 × 10^5^ PFU to 0 PFU. Positive-sense viral RNA was detected using N3 primers, with probe and reaction conditions previously described (34).

### Histopathological analysis.

Sections of tissue from all animals were fixed in 10% neutral buffered formalin and processed to paraffin wax, and 3- to 5-μm-thick sections were cut and stained with hematoxylin and eosin (H&E). Tissues were examined by light microscopy and evaluated subjectively. Slides from age-matched unchallenged animals were evaluated to establish the nature of background, incidental lesions. Imagic IMS software was used to capture and store digital images.

### DPP4 receptor histology and immunohistochemistry.

The upper respiratory tract, including the nasal cavity (respiratory and olfactory areas), nasopharynx, and larynx, was subjected to a slow decalcification process before being processed to paraffin wax, and 4-μm-thick sections were cut and stained with H&E. A total of 24 paraffin-embedded tissue blocks were produced. Each lung block included two pieces of tissue to ensure the presence of large bronchi and small bronchioles and alveoli. MERS-CoV IHC staining was performed by subjecting the tissue sections to heat-induced epitope retrieval using ER1, a citrate-based buffer (catalog number AR9961; Leica Biosystems), for 20 min at 95°C. A mouse monoclonal antibody specific for MERS-CoV nucleocapsid antibody (Sino Biological) diluted 1:1,000 was applied and incubated for 15 min, and the Leica polymer refine kit (Leica Biosystems) was used for visualization. For DPP4 IHC staining, the tissue sections were subjected to heat-induced epitope retrieval using ER1, a citrate-based buffer (catalog number AR9961; Leica Biosystems), for 20 min at 95°C before applying a goat polyclonal anti-human DPP4 antibody (R&D Systems) diluted 1:250 and incubated for 15 min. A rabbit anti-goat secondary IgG antibody (Abcam, UK) was then applied for 8 min before using the Leica Intense R detection kit (Leica Biosystems) for visualization.

### Cell type determination.

Blood samples or single-cell suspensions of lungs and spleens were used for immunological analysis. Red blood cells were lysed using RBC lysis buffer (BD Biosciences), and the remaining leukocytes were stained using three sets of five to seven antibody-bound fluorescent stains to identify cell phenotypes and activation status by flow cytometry. The following combinations of mouse anti-human fluorescent-antibody stains were used: CD3 (SP34-2), CD8 (LT8), CD56 (B159), CD69 (FN50), CD20 (Bly1), CD16 (3G8), and gamma delta T cells (B1) for lymphocytes and CD11c (SHCL3), CD14 (M5E2), CD16 (3G8), CD54 (HCD54), CD163 (GHI/61), CD64 (10.1), CD66b (G10F5), CD80 (2D10), CD40 (5C3), and HLA-DR (L243) for monocytes/macrophages and neutrophils (BD Bioscience, BioLegend, and AbD Serotec). All samples were fixed in 4% paraformaldehyde for 48 h at 4°C and analyzed by flow cytometry (BD FACSCanto II, using the DIVA data package) within 72 h of staining. Basic cell types (lymphocytes, monocytes, and granulocytes) were determined by forward and side scatter, and cellular debris was differentiated from intact cells by nuclear staining. Neutrophils were differentiated from monocytes by the intensity of CD11c and CD14 staining.

### Serological analysis.

Ninety-six-well tissue culture plates were coated with ∼1 × 10^5^ PFU/mL of live MERS-CoV (either strain EMC/2012 or England 1) in coating buffer and incubated overnight at 4°C. The plates were washed in PBS and 0.05% Tween 20. Wells were blocked with 100 μL 5% (wt/vol) skimmed milk powder in PBS and 0.05% Tween 20 at 37°C for 60 min and washed three times in PBS and 0.05% Tween 20. Plasma samples were added at a 1:50 dilution in Blotto, in duplicate. Each plate included naive marmoset plasma as a negative control. The plate was incubated for 2 h at 37°C and washed three times with PBS and 0.05% Tween 20. One hundred microliters of a 1:1,000 dilution of isotype-specific goat anti-human IgG-horseradish peroxidase (HRPO) conjugates (Oxford Biotechnology) was added to each well, and the plate was incubated for 1 h at 37°C. The plate was washed a further three times in PBS-Tween, and 100 μL of the 3,3′,5,5′-tetramethylbenzidine (TMB) substrate was then added to each well for 20 min before adding stop solution (HCl and H_2_SO_4_). The plates were read at 450 nm.

### Plaque reduction neutralization test.

Vero E6 cells were maintained in Dulbecco’s modified Eagle medium (DMEM) supplemented with 10% fetal bovine serum (FBS) and 100 U/mL of penicillin-streptomycin. The assay was performed in 24-well tissue culture plates (Corning Costar; Thermo Scientific) in a biosafety level 3 facility. Next, 1:10 and 1:50 dilutions of each serum sample (pre- and postchallenge) were heat treated at 56°C for 30 min to inactivate complement and then incubated with 1,200 PFU of virus/mL L-15 medium for 1 h at 37°C. Two-hundred-microliter virus-serum mixtures were added in duplicate onto preformed Vero E6 cell monolayers and incubated for 1 h at RT with gentle rocking. The cell monolayer was then overlaid with 1% agarose in L-15 medium and incubated for 7 days, at which time the plates were fixed and stained. Antibody titers were defined as the highest serum dilution that resulted in a ≥90% (PRNT_90_) or a >50% (PRNT_50_) reduction in the number of virus plaques compared to prechallenge samples.

### Statistical analysis.

Immunological, hematological, and clinical chemistry parameters in the blood were compared for each animal prechallenge and with blood collected postmortem using one-way analysis of variance (ANOVA) (with some data transformed by log_10_ to ensure a normal distribution). The time and duration of fever were analyzed by Pearson’s correlation.

### Statistics.

All statistical analysis was performed using GraphPad Prism v8.0.1. Comparative analysis of immunology, hematology, blood chemistry, and aerobiology data was performed using ANOVA or a *t* test on transformed data, *Y* = log(*Y*), to ensure a normal distribution. ANOVA was also performed on untransformed virological titers.
